# Characteristics and mechanisms of mosaicism in prenatal diagnosis cases by application of SNP array

**DOI:** 10.1186/s13039-023-00648-y

**Published:** 2023-07-03

**Authors:** Lili Zhou, Huanzheng Li, Chenyang Xu, Xueqin Xu, Zhaoke Zheng, Shaohua Tang

**Affiliations:** Center of Prenatal Diagnosis, Wenzhou Central Hospital, Wenzhou, 325000 People’s Republic of China

**Keywords:** Mosaicism, SNP array, Mechanism, Prenatal diagnosis

## Abstract

**Background:**

With the application of chromosome microarray, next-generation sequencing and other highly sensitive genetic techniques in disease diagnosis, the detection of mosaicism has become increasingly prevalent. This study involved a retrospective analysis of SNP array testing on 4512 prenatal diagnosis samples, wherein the characterization of mosaicism was explored and insights were gained into the underlying mechanisms thereof.

**Results:**

Using SNP array, a total of 44 cases of mosaicism were identified among 4512 prenatal diagnostic cases; resulting in a detection rate of approximately 1.0%. The prevalence of mosaicism was 4.1% for chorionic villus sample, 0.4% for amniotic fluid, and 1.3% for umbilical cord blood. Of these cases, 29 were mosaic aneuploidy and 15 were mosaic segmental duplication/deletion. Three cases of mosaic trisomy 16 and three cases of mosaic trisomy 22 were diagnosed in the CVS samples, while four cases of mosaic trisomy 21 were detected in amniotic fluid and umbilical cord blood samples. The distribution pattern of mosaicism suggested trisomy rescue as the underlying mechanism. Structurally rearranged chromosomes were observed, including three cases with supernumerary marker chromosomes, three cases with dicentric chromosomes, and one case with a ring chromosome. All mosaic segmental duplication/deletion cases were the result of mitotic non-disjunction, with the exception of one case involving mosaic11q segmental duplication.

**Conclusion:**

Improved utilization of SNP arrays enables the characterization of mosaicism and facilitates the estimation of disease mechanisms and recurrence.

## Background

Mosaicism occurs when a single fertilized egg develops into an embryo containing two or more populations of cells with distinct genotypes [1]. According to the variation types, chromosomal abnormalities can be divided into aneuploidy, polyploidy, segmental duplications/deletions, translocations, inversions, ring chromosomes, isochromosomes, etc. The most frequent type of aneuploidy mosaicism is gonosomal aneuploidy, while mosaicisms involving abnormal chromosome structure are relatively uncommon. The clinical phenotype of mosaicism is variable, ranging from mild mental retardation in genetic syndromes such as Pallister-Killian syndrome and Ito, to embryonic lethality [2–4].

During prenatal diagnosis, the incidence of mosaicism in chorionic villus samples is about 1%~2%. However, in most cases, it is confined placental mosaicism (CPM) accounting for 86.5%, while true fetal mosaicism (TFM) accounts for only 13.5% [5, 6]. In amniotic fluid samples, the incidence of TFM is about 0.1%~0.3%.

Both fetal mosaicism and placental mosaicism can lead to prenatal or perinatal complications [7, 8]. However, the mechanisms behind these conditions have vastly different impacts on fetal development. In cases of mosaic aneuploidy originating from meiosis, the aneuploid constitution likely occurs in the very early stages of embryo development, where correct chromosome number might be vital [9, 10]. Conversely, mitotic-originated cases may proceed with normal early cleavage but could potentially affect a subset of tissues. Differentiation between a mitotic and meiotic origin of trisomies is necessary for determining recurrence risks and for proper counseling; this is because mosaic trisomy arising from meiotic non-disjunction is associated with a higher risk of recurrence, especially in younger women [11].

Mosaicism can be identified by various methods, including chromosome karyotyping, fluorescence in situ hybridization, chromosome microarray, and next-generation sequencing. However, single nucleotide polymorphism (SNP) array analysis offers several advantages over traditional chromosome karyotyping for detecting mosaicism. With SNP array analysis, a large number of cells can be detected simultaneously and culture bias can be eliminated by analyzing interphase cells [12, 13]. For cases of complex mosaicism, SNP array can provide crucial information on the characteristics of the mosaicism (including content, origin, and mechanism), which are essential for accurate fetal prognosis assessment and genetic counselling.

Here, we conducted a study on 4512 pregnant women referred for prenatal diagnosis, utilizing genome-wide SNP arrays and karyotype analysis to investigate the characteristics and mechanisms of mosaicism.

## Results

### Distribution of mosaicism by sample type and mosaic type

A total of 44 cases of mosaicism were initially detected among the 4512 patients using genome-wide SNP array; the overall prevalence of mosaicism was 1.0% (44/4512 cases). Of these, 21 cases were identified from chorionic villus sample, 11 cases from amniotic fluid, and 12 cases from umbilical cord blood. The prevalence of mosaicism was 4.1% for chorionic villus sample, 0.4% for amniotic fluid, and 1.3% for umbilical cord blood. In this cohort, 29 cases were diagnosed as mosaic aneuploidy and 15 cases were mosaic segmental duplication/deletion (Tables 1 and 2). Among the cases exhibiting mosaic aneuploidy, fifteen were diagnosed as mosaic trisomy, one as mosaic monosomy, six as gonosomal aneuploidy mosaicism, six as double trisomy, and one as near-diploid. By comparing the percentage of mosaicism in each case between the array data and conventional karyotype analysis, a total of 18 mosaic aneuploidy cases showed differential results with a discordance rate of 66.7% (18/27).

**Table 1:** Results of 29 cases with mosaic aneuploidy

Case No	Specimen	Type of aneuploidy	Mosaic% by SNP array	Karyotype	Mosaic% by karyotype	Origin	Indication
1	Villus	T 4	30	–	–	Mitosis	IFD
2	UCB	T 8	20	47,XY,+8[3]/46,XY[16]	15	Mitosis	MR in mother
3	Villus	T 8	80	47,XX,+8[8]/46,XX[2]	80	Mitosis	IFD
4	Villus	T 15	60	46,XY,+15,rob(15;15)(q10;q10)	100	Mitosis	IFD
5	Villus	T 16	20	47,XX,+16[8]/46,XX[2]	80	M I	IFD
6	Villus	T 16	30	47,XX,+16[3]/46,XX[7]	30	M I	IFD
7	Villus	T 16	20	47,XY,+16[3]/46,XY[7]	30	M I	IFD
8	AF	T 18	80	47,XX,+18[38]/46,XX[8]	82.61	M II	high T18 risk
9	AF	T 21	20	46,XY,+21,rob(21;21)(q10;q10)[3]/46,XY[16]	15	Mitosis	AMA
10	AF	T 21	20	47,XY,+21[1]/46,XY[49]	2	M II	abnormal NIPT
11	AF	T 21	70	47,XX,+21[32]/46,XX[9]	76.19	M II	high T 21 risk
12	UCB	T 21	60	45,XY,rob(15;21)(q10;q10)	0	Mitosis	ventriculomegaly
13	Villus	T 22	80	47,XX,+22[8]/46,XX[2]	80	Mitosis	IFD
14	Villus	T 22	30	47,XX,+22[5]/46,XX[5]	50	M II	IFD
15	Villus	T 22	15	47,XX,+22[4]/46,XX[7]	36.36	M I	IFD
16	Villus	T 2	100	47,XY,+2	100	Meiosis	IFD
		T 20	60		0	Mitosis	
17	Villus	T 7	50	48,XX,+7,+21[11]/47,XX,+21[2]	85.71	Mitosis	IFD
		T 21	70		100	M I	
18	Villus	T 13	20	46,XY,rob(13;14)(q10;q10),+rob(13;14)(q10;q10)	100	Mitosis	IFD
		T 14	20		100	Mitosis	
19	Villus	T 13	15	48,XY,+13,+14[4]/46,XY[1]	80	M II	IFD
		T 14	20		80	M I	
20	Villus	T 14	30	48,XY,+14,+20	100	Mitosis	IFD
		T 20	30		100	Mitosis	
21	Villus	T 18	100	48,XX,+18,+22[2]/47,XX,+18[8]	100	M II	IFD
		T 22	15		20	M I	
22	Villus	T 13	15	50,XX,+13,+18,+19,+21	100	M II	IFD
		T 18					
		T 19					
		T 21					
23	Villus	M 14	60	–	–	Mitosis	IFD
24	AF	X	20	45,X[6]/46,XX[13]	30	Mitosis	abnormal NIPT
25	AF	X	20	45,X[7]/46,XX[14]	31.82	Mitosis	AMA
26	AF	X	50	45,X[16]/47,XXX[6]	74	Mitosis	AMA
		XXX	50		26		
27	UCB	X	50	45,X[7]/46,XY[39]	15.22	Mitosis	abnormal NIPT
28	AF	XXY	60	47,XXY[4]/46,XY[17]	18.18	Mitosis	AMA
		XY	40		81.82		
29	Villus	XXY	20	47,XXY[14/46,XY[6]	40	M I	IFD
		XY	80		60

**Table 2:** Results of 15 cases with mosaic segmental duplication/deletion

Case No	Specimen	SNP array	Type	Size (Mb)	Karyotype	Indication
30	UCB	arr 1q31.3q44×2~3	Dup	52.24	46,XX,add(1)(q44)[6]/46,XX[20]	Neural tube malformation
31	UCB	arr 2p25.2p24.2×1~2	Del	11.42	46,XY,del(2)(p24.2p25.2)[6]/46,XY[14]	Unknown
32	AF	arr 15q11.2q13.1×2~6	Dup	7.61	48,XX,+2mar[30]/47,XX,+mar[28]/46,XX[7]	AMA
33	AF	arr 21q11.2q21.1×2~4	Dup	4.18	47,XY,+mar[13]/46,XY[15]	High T 21 risk
34	UCB	arr (12)×2~3,	Dup	133.56	47,XX,+mar	Choroidal fissure cyst
		12p11.23p11.1×3	Dup	7.23		
35	UCB	arr Xp22.33p22.31×1	Del	8.46	46,X,psu idic(X)(p22.3)[38]/45,X[3]	Unknown
36	AF	arr Yp11.32q11.222×0~2,	Del	2.9	45,X[20]/46,X,idic(Y)(q11.22)[5]/46,XY[18]	Abnormal NIPT
		Yq11.222q11.223×0	Dup	20.76		
37	UCB	arr 4p16.3p14×1,	Del	37.12	45,XX,dic(4;22)(p11;p11.2)	UMM
		4p14q35.2×2~3,	Dup	153.36		
		(22)×2~3	Dup	34.17		
38	UCB	arr 5p15.33p13.3×1,	Del	33.34	46,XX,r(5)(p13q35)	Intrauterine growth retardation
		5p13.3q35.3×2~3	Dup	147.34		
39	UCB	arr 1q32.2q44×3,	Dup	38.79	46,XY,add(6)(p25)	Enlarged posterior cranial fossa
		3q11.1q29×2~3	Dup	99.87		
40	Villus	arr Xp22.33q21.33×1~2,	Del	95.60	45,X	IFD
		Xq21.33q28×1	Del	59.33		
41	UCB	arr 11q23.3q25×2~3	Dup	15.92	46,XX	Enlarged pelvis
42	UCB	arr 11q13.4q25×2~3	Dup	63.24	46,XX	PCF
43	Villus	arr 11q14.3q24.1×2~3,	Dup	31.54	46,XY	IFD
		11q24.1q25×1~2	Del	13.27		
44	Villus	arr 3p26.3p26.1×1,	Del	4.98	46,XY	IFD
		3p26.1p21.31×2~3,	Dup	39.78		
		6p25.3p22.1×2~3	Dup	28.02		

*UCB* Umbilical cord blood; *AF* Amniotic fluid; *T* Trisomy; *M* Monosomy; *MI* Meiosis I; *MII* Meiosis II; *IFD* Intrauterine fetal death; *PCF* Posterior cranial fossa communicates with the lateral ventricle; *MR* Mental retardation; *AMA* Advanced maternal age; *UMM* Ultrasound multiple malformation

### Mosaic trisomy

Fifteen cases of mosaic trisomy were detected. Comparison between the array and karyotype analysis indicated that the eight cases exhibited concordant results (a 10% deviation was deemed to be within acceptable limits), while six cases showed discordance; there was a failure of cultural testing in one case of mosaic trisomy 4.

**Fig. 1:**
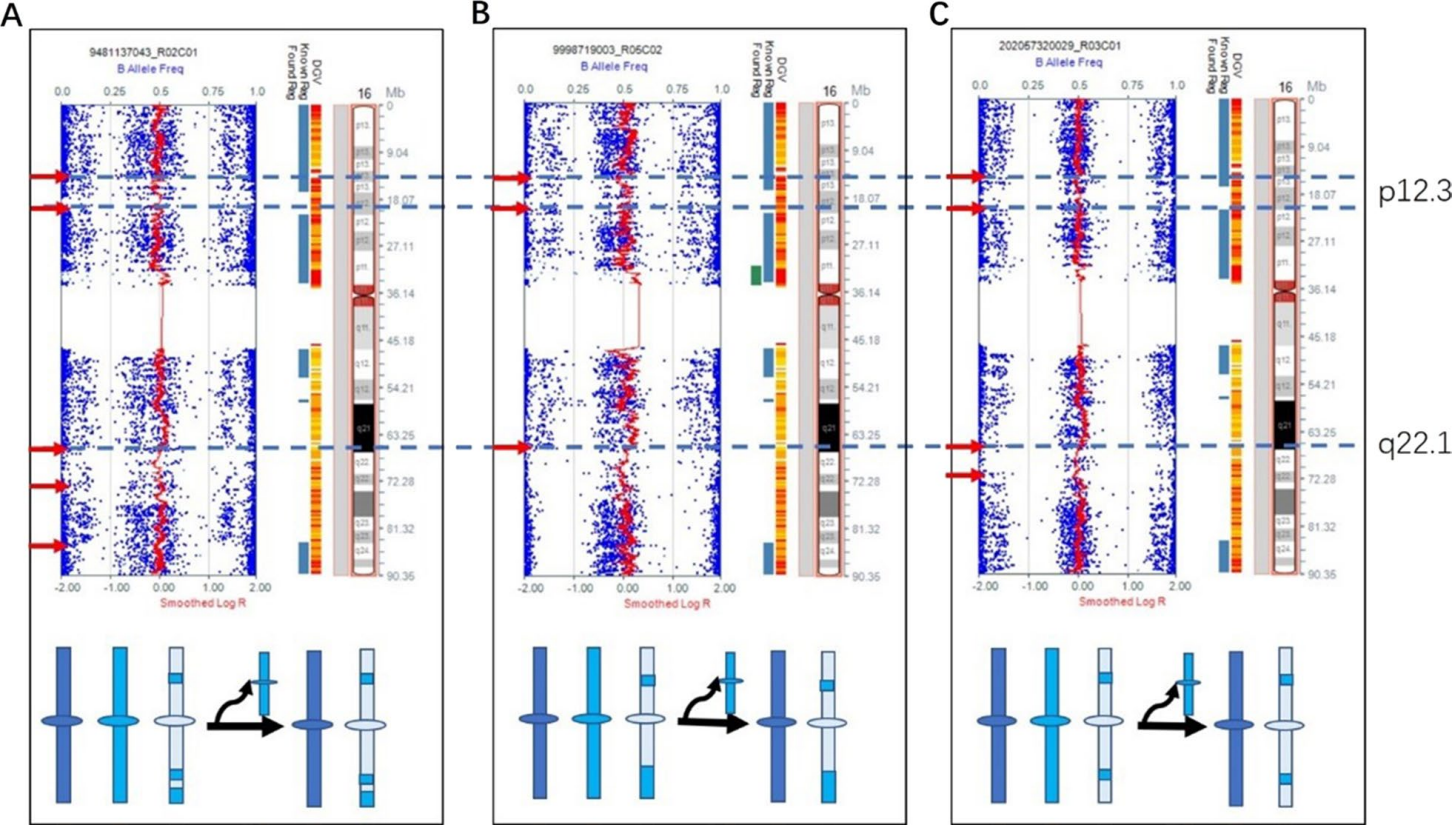
SNP array results and mechanisms for mosaic trisomy 16. The log R ratio indicates an increase in copy number, between two and three copies. Additional shifts in the B allele frequency are observed, corresponding to a shift in B allele frequency from 0% towards 33% (in the case of AA in the euploid cell line and AAB in the trisomic cell line), and a shift from 100% toward 66% (in the case of BB in the euploid cell line and ABB in the trisomic cell line). The additional haplotypes are visible near the centromere, consistent with a meiosis I nondisjunction. The crossovers were in 16p12.3 and 16q22.1, indicating that these loci were hot spot of recombination. **A** Mosaic trisomy 16 in case 5 arose from meiosis I with the presence of five visible crossovers. The proportion of cells exhibiting mosaic trisomy 16 was 20%. **B** Mosaic trisomy 16 in case 6 arose from meiosis I with 3 crossovers. The proportion of cells exhibiting mosaic trisomy 16 was 30%. **C** Mosaic trisomy 16 of case 7 arose from meiosis I with 4 crossovers. The proportion of cells exhibiting mosaic trisomy 16 was 20%

**Fig. 2:**
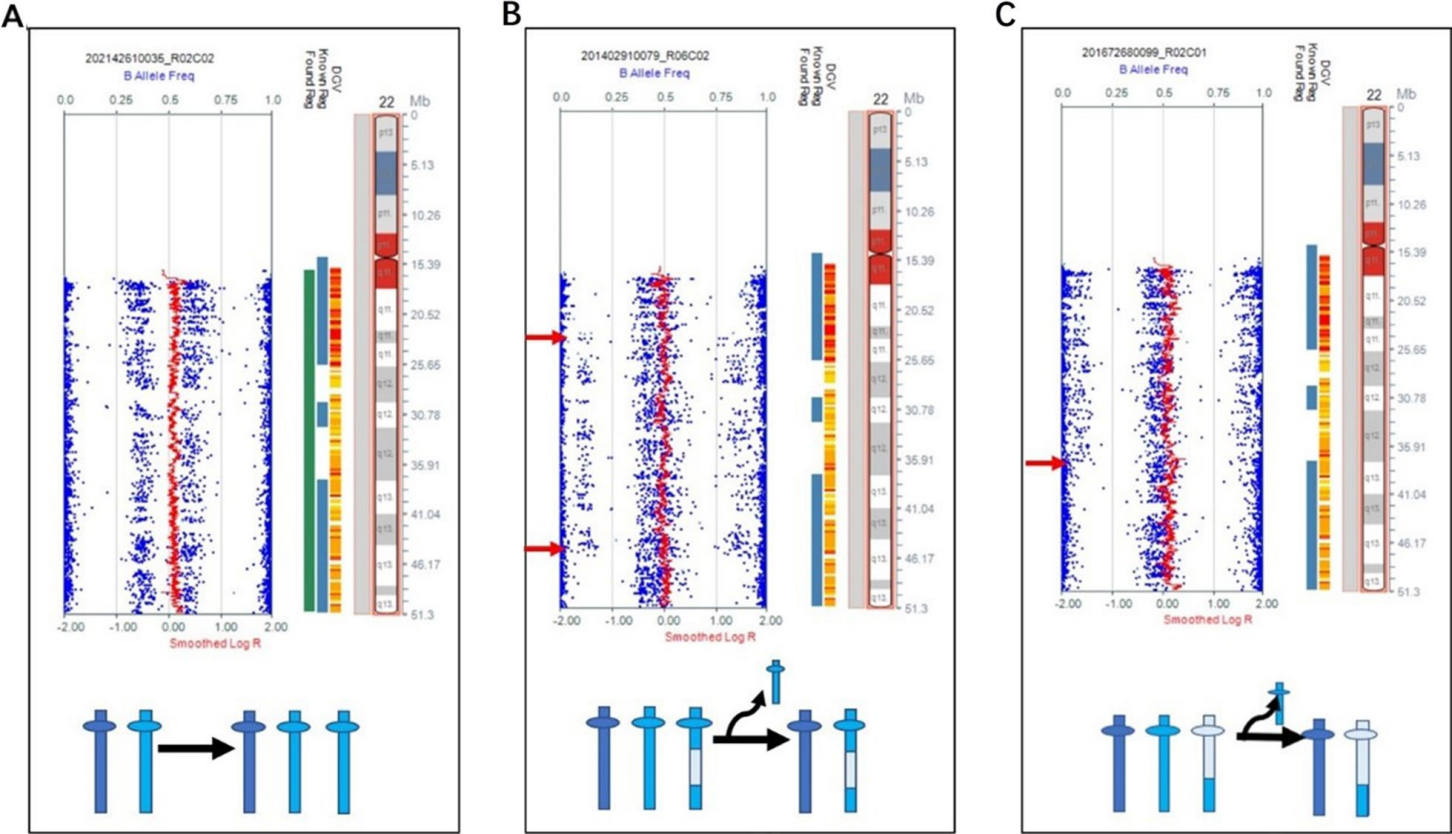
SNP array results and mechanisms for mosaic trisomy 22. **A** Mosaic trisomy 22 in case 13 arose from mitotic nondisjunction without crossover, with mosaic trisomy 22 cells accounting for 80% of the total. A mitotic origin was suggested by the absence of a third haplotype, indicated on the SNP array by shifts in the B allele frequency, corresponding to a shift in B allele frequency from 50% towards 33%, and a shift from 50% toward 66%. **B** Mosaic trisomy 22 in case 14 arose from meiotic II nondisjunction with a frequency of 30%. The additional haplotypes are observable near the telomeres, but not at the centromere, which is consistent with a meiosis II origin, where sister chromatids undergo non-disjunction with two crossovers. **C** Mosaic trisomy 22 in case 15 arose from meiotic I nondisjunction with a frequency of 15%. The additional haplotypes are visible proximal to the centromere with one crossover

In our cohort, seven cases of mosaic trisomy arose by mitotic non-disjunction (Figs. 1 and 2). Four cases of mosaic trisomy arose by meiotic I non-disjunction, including one case of mosaic trisomy 22 and three cases of mosaic trisomy 16, with additional haplotypes visible near the centromere. Those three cases of mosaic trisomy 16 were determined by the visible patterns of recombination with 3–5 crossovers (Fig. 1). The crossovers were in 16p12.3 and 16q22.1, indicating that these loci were hot spots of recombination. Four cases arose by meiotic II non-disjunction, including one case each of mosaic trisomy 18 and 22, as well as two cases of mosaic trisomy 21; these cases exhibited additional haplotypes in close proximity to the telomeres.

Three cases of mosaic trisomy 16 and three cases of mosaic trisomy 22 were diagnosed in the CVS samples, both of which can be lethal during the first trimester. On the other hand, four cases of mosaic trisomy 21 were detected in amniotic fluid and umbilical cord blood samples, which can be better tolerated by the developing embryo. The distribution pattern of mosaicism suggested trisomy rescue as the underlying mechanism.

### Gonosomal aneuploidy mosaicism

Six cases of gonosomal aneuploidy mosaicism were detected. Comparison between the SNP array and karyotype analyses revealed differential results for the gonosomal aneuploidy mosaicism cases, except for two cases of 45,X/46,XX. Overall, the mosaic frequencies of gonosomal aneuploidy mosaicism in the array data were unclear.

All mosaic gonosomal aneuploidies arose by mitotic non-disjunction, with the exception of one case of 47,XXY/46,XY which arose from meiotic I non-disjunction. With the exception of this particular case resulting in intrauterine fetal death during the first trimester, all other cases survived until the second or third trimester. This suggests that chromosome X monosomy is associated with less embryonic lethality than euchromosome monosomy when non-disjunction occurs mitotically.

### Mosaic double trisomies and near-diploid

Six cases of mosaic double trisomies were identified. Comparison between the array and karyotype analyses revealed differential results for the double trisomy cases, except for one case of +18/+22. All cases resulted in intrauterine fetal death during the first trimester, indicating that these mosaic double trisomies cannot be tolerated by the embryo.

**Fig. 3:**
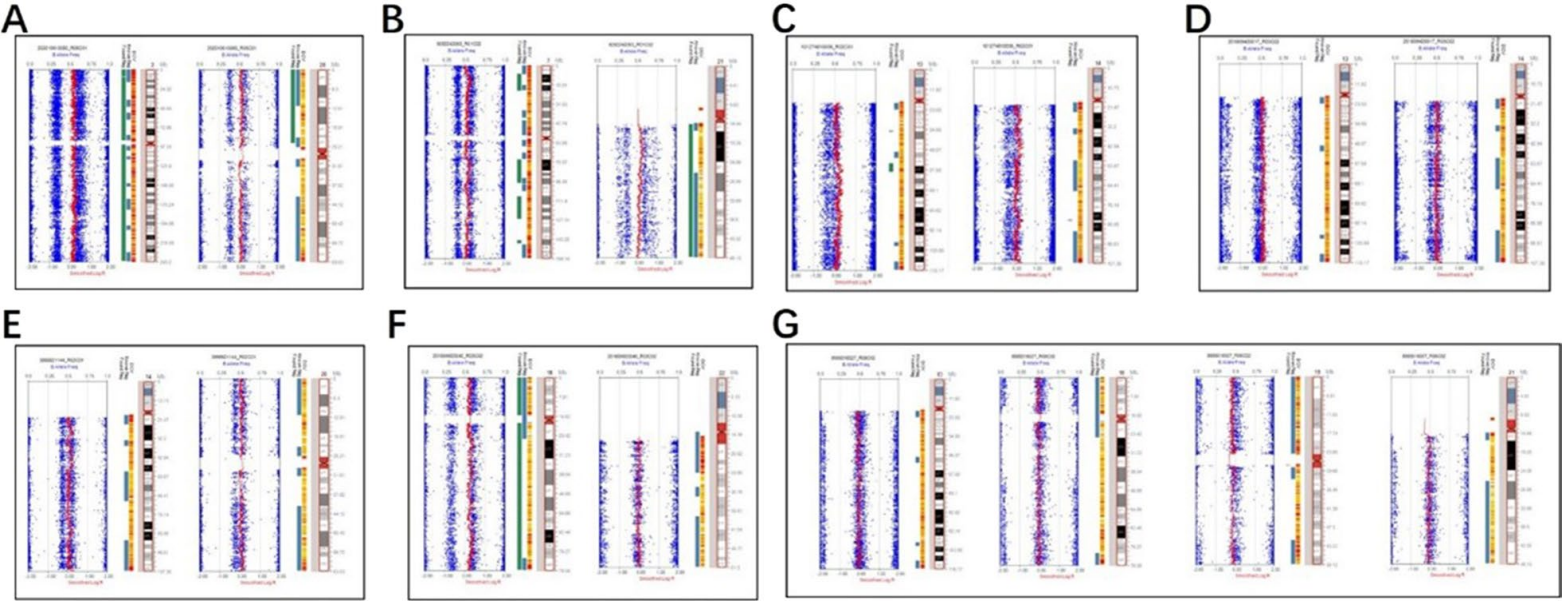
SNP array of six cases with mosaic double trisomies and one case with mosaic near-diploid. The mechanisms of mosaic double trisomies and near-diploid are intricate; with the possibility of either congruous or incongruous origins for the affected chromosomes. The occurrence of errors in **A**, **B**, **D**, and **F** is characterized by varying mosaic frequencies across different chromosomes. The origin of **C** and **E** were from mitotic non-disjunction errors. **G** was from a meiosis II non-disjunction error with simultaneous occurrence at the same mosaic frequencies

One case with mosaic near-diploid of +13/+18/+19/+21 was detected. This rare abnormality arose from simultaneous meiosis II, in which four chromosomes exhibited the same mosaic frequency of 15%; however, the result of karyotype analysis indicated a non-mosiac near-diploid with 50,XX,+13,+18,+19,+21 (Fig. 3).

### Mosaic segmental duplication/deletion cases

Among the fifteen cases of mosaic segmental duplication/deletion, twelve cases involved euchromosomes and three cases involved sex chromosomes. In terms of mosaic types, seven cases were characterized by duplication, two cases by deletion and six cases by a combination of both. Nevertheless, three cases resulted in intrauterine fetal death during the first trimester, while the remaining twelve cases survived until the second or third trimester. Among these twelve cases, seven cases (58.3%) with euchromosomes exhibited abnormal ultrasonographic findings. Thus, mosaic segmental duplication/deletion resulted in lower embryonic lethality than mosaic aneuploidy. Additionally, mosaic segmental duplication/deletion had a high incidence of ultrasound malformations, especially in cases of mosaic segmental duplication.

**Fig. 4:**
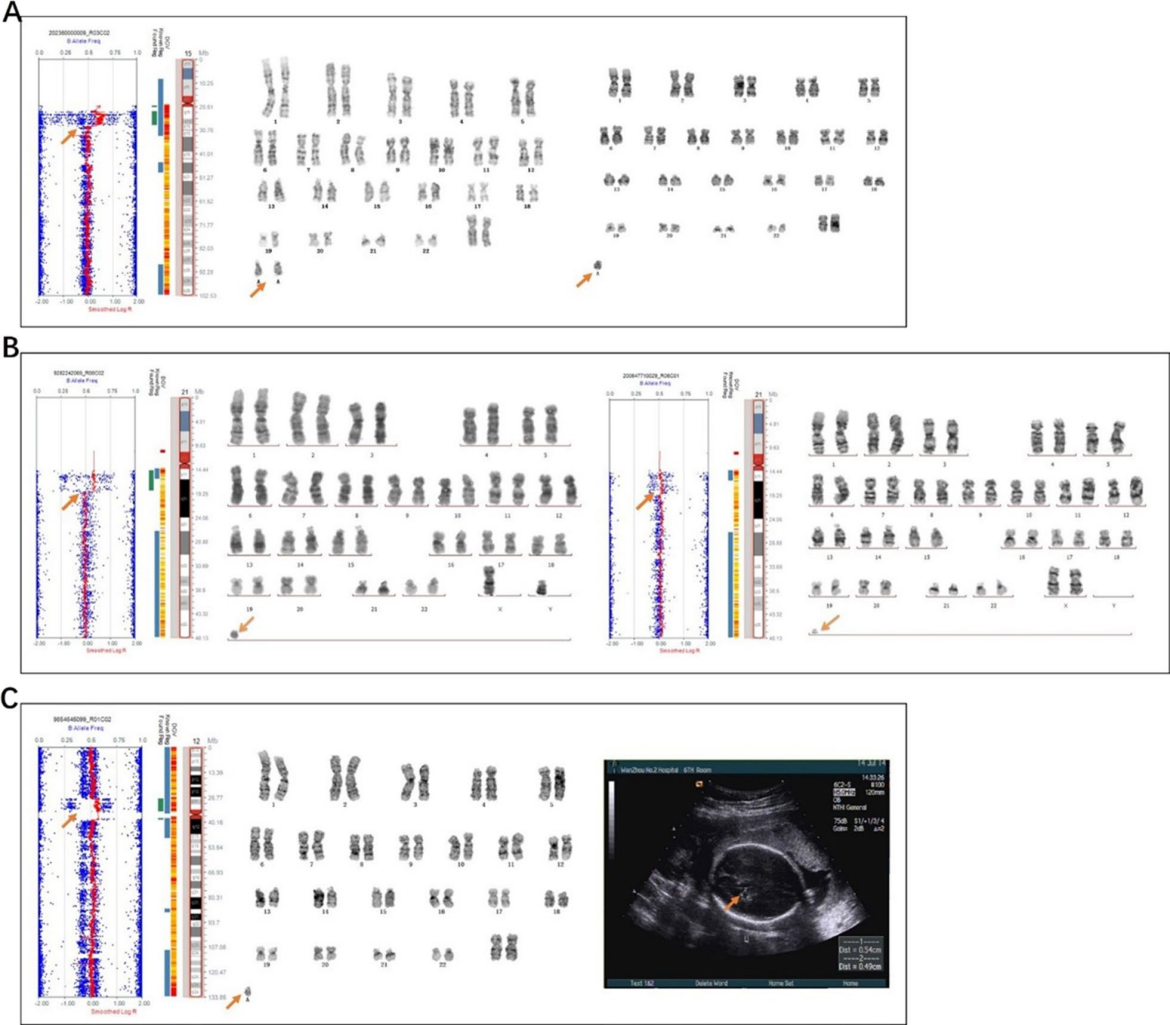
SNP array and karyotype results of small supernumerary marker chromosomes. **A** SNP array revealed 15q11.2q13.1×2~6 and karyotype was 48,XX,+2mar[30]/47,XX,+mar[28]/46,XX[7] for case 32. **B** SNP array showed 21q11.2q21.1×2~4 and karyotype was 47,XY,+mar[13]/46,XY[15] for case 33. In addition, the SNP array of the mother was 21q11.2q21.1×2~3 and her karyotype was 47,XX,+mar[10]/46,XX[39]. **C** SNP array was (12)×2~3, 12p11.23p11.1×3 and karyotype was 47,XX,+mar for case 34. The presence of a choroidal fissure cyst malformation was detected via ultrasound examination

**Fig. 5:**
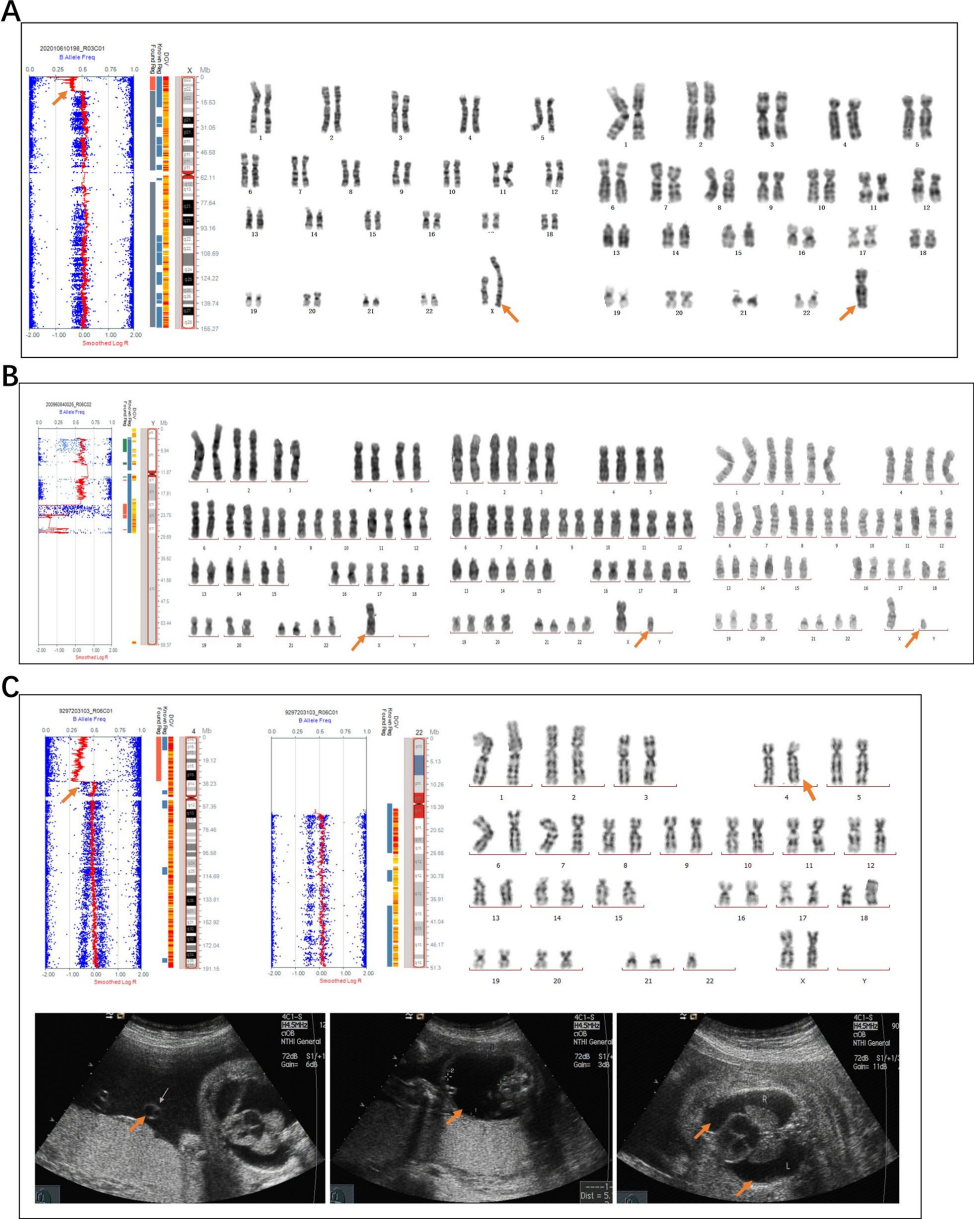
SNP array and karyotype of dicentric chromosomes. **A** SNP array showed Xp22.33p22.31×1 and karyotype was 46,X,psu idic(X)(p22.3)[38]/45,X[3] for case 35. **B** SNP array showed Yp11.32q11.222×0~2, Yq11.222q11.223×0 and karyotype was 45,X[20]/46,X,idic(Y)(q11.22)[5]/46,XY[18] for case 36. **C** SNP array showed 4p16.3p14×1, 4p14q35.2×2~3, (22)×2~3 and karyotype was 45,XX,dic(4;22)(p11;p11.2) for case 37. Multiple ultrasound malformations were observed in case 37, including a single umbilical artery, ascites, and bilateral pleural effusion

**Fig. 6:**
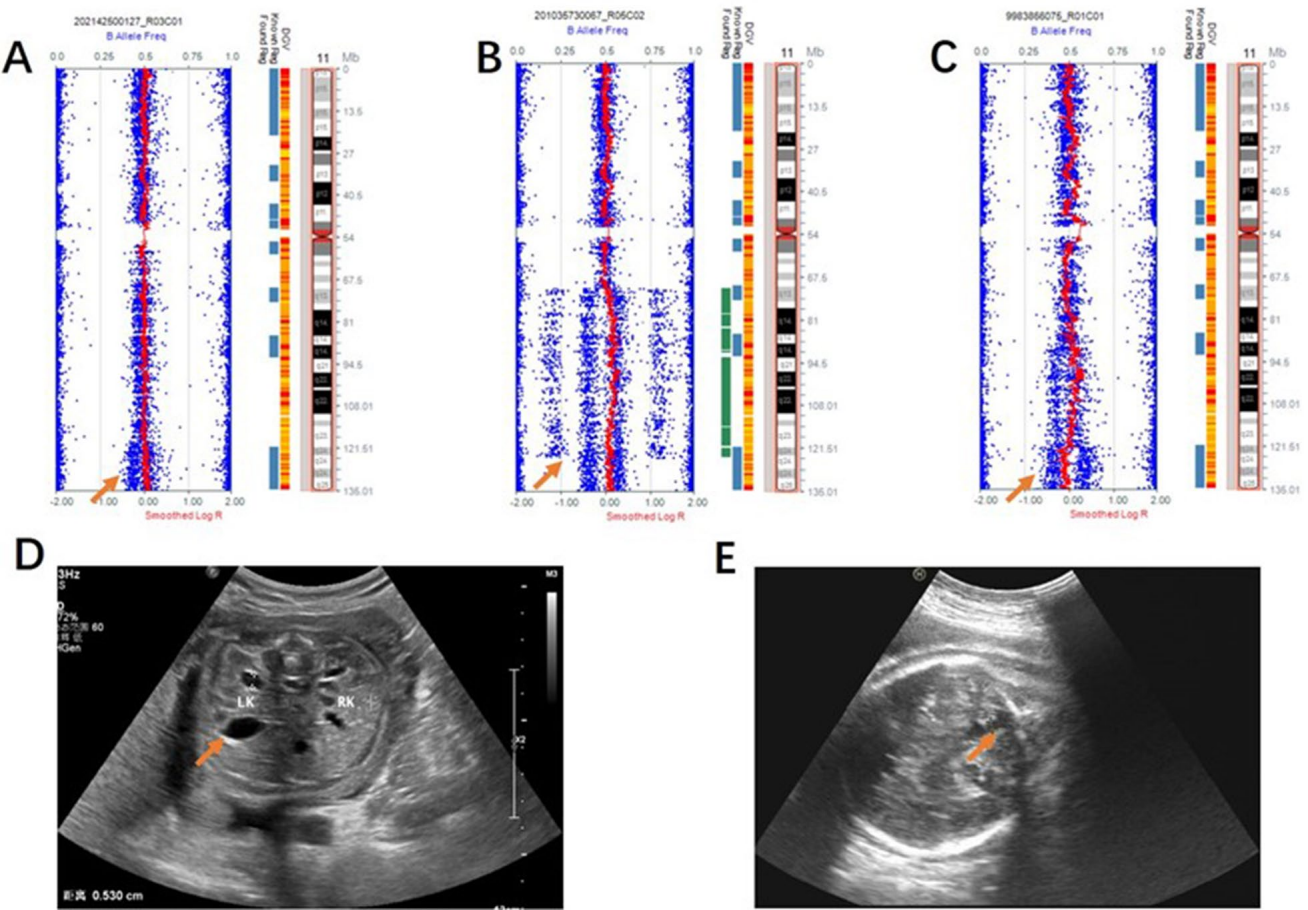
SNP array and ultrasound malformations of mosaic 11q segmental duplication. **A** Case 41 arose by mitotic non-disjunction with mosaic duplication of 11q23.3q25. **B** Case 42 arose by meiotic I non-disjunction with mosaic duplication of 11q13.4q25. **C** Case 43 arose by mitotic non-disjunction with mosaic duplication of 11q14.3q24.1 combined with mosaic deletion of 11q24.1q25. **D** Enlarged pelvis malformation was observed on ultrasound for case 41. **E** The ultrasound malformation of case 42 was a posterior cranial fossa communicating with the lateral ventricle

In our cohort, there were three cases of small supernumerary marker chromosomes (sSMC), three cases of dicentric chromosomes, and one case of a ring chromosome (Figs. 4 and 5). Further, there were three cases of mosaic 11q segmental duplication with normal karyotypes (Fig. 6).

Here, eleven cases exhibited abnormal karyotypes and four cases displayed normal karyotypes. A comparison between the array and karyotype analyses revealed that only two cases showed concordant results, which involved simple mosaic duplication or deletion greater than 10 Mb. The remaining thirteen cases had differential results, with a coincidence rate of only 13.3%.

## Discussion

With the application of chromosome microarray, next-generation sequencing and other highly sensitive genetic techniques in disease diagnosis, the detection of mosaicism has become increasingly prevalent. In our study, a total of 44 cases of mosaicism were initially detected among the 4512 patients using genome-wide SNP array; the overall prevalence of mosaicism was 1.0% (44/4512 cases).

For different prenatal diagnostic samples, mosaic aneuploidy was found in about 1–2% of chorionic villus samples and 0.2% of amniocentesis samples obtained for prenatal diagnosis [14]. In our survey, the frequency of mosaicism was 4.1% in chorionic villus samples, 0.4% in amniotic fluid samples, and 1.3% in umbilical cord blood samples. Thus, the frequency of these events exceeded our initial expectations. We hypothesize that mosaicism may be more prevalent than previously anticipated, particularly in fetal tissue obtained from abortions. The high frequency of mosaicism observed in chorionic villus samples may be subject to bias due to the potential presence of confined placental mosaicism, which have not been excluded by the second prenatal testing. The incidence of mosaicism is relatively low in karyotype analysis of the umbilical cord blood, and there is insufficient large-scale research data available in this area. In our paper, we found that only three cases of the umbilical cord blood were mosaic aneuploidy, while nine cases were mosaic segmental duplication/deletion. These findings suggest that the incidence of mosaicism in umbilical cord blood may be higher than previously reported, and structurally rearranged chromosomes of mosaicism were readily observed in umbilical cord blood.

We calculated the percentage of mosaicism in each case from the array data and compared it with the findings from karyotype analysis. The results of eighteen cases with mosaic aneuploidy exhibited a differential outcome, with a discordance rate of 66.7% (18/27). Although mosaicism can be identified cytogenetically, the metaphase may provide a biased view due to culture influences or specific abnormalities such as Pallister Killian syndrome [4]. The SNP array presents advantages in detecting mosaicism, as it eliminates culture bias and provides insight into underlying mechanisms. However, it is not suitable for the detection of gonosomal mosaic aneuploidies. SNP array tests mixtures of DNA, thus it cannot confirm the actual type and frequencies of gonosomal mosaic aneuploidy. Six cases of gonosomal mosaic aneuploidy were detected in this study, and comparison between the array and karyotype analyses revealed discrepancies in their results, except for two cases of 45,X/46,XX. We hypothesized that the SNP array tests DNA mixtures, thus it is unable to confirm the precise types and frequencies of gonosomal mosaic aneuploidy.

In our study, SNP array analysis revealed a total of fifteen mosaic segmental duplication/deletion cases, including three cases with small supernumerary marker chromosomes (sSMCs), three cases with dicentric chromosomes, and one case with a ring chromosome. All mosaic segmental duplication/deletion cases were the result of mitotic non-disjunction, with the exception of one case involving mosaic11q segmental duplication. It is known that those structurally rearranged chromosomes often lead to mosaicism during mitotic disjunction. The clinical consequences of instability may surpass those related to the chromosomal abnormalities themselves [15–18]. For instance, the karyotype was 45,XX,dic(4;22)(p11;p11.2) for case 37, while SNP array showed 4p16.3p14×1, 4p14q35.2×2~3, (22)×2~3. Two thirds of retained dicentrics undergo epigenetic centromere inactivation, followed by a breakage event results in the formation of two monocentric parental chromosomes [19]. The SNP array analysis revealed additional genomic abnormalities beyond the deletion of the short arm of chromosome 4, providing insights into the pathogenic cause and underlying mechanism.

Previous work has demonstrated that there is a chromosome-specific bias in the ratio of meiotically to mitotically occurring non-disjunctions [9, 10]. For instance, the vast majority of trisomy 16 cases are linked to errors occurring during maternal meiotic I, while trisomy 18 is frequently linked to meiotic II errors and both trisomy 21 and trisomy 22 are commonly linked to meiotic I errors [20]. Our findings are consistent with prior research. Four cases of mosaic trisomy arose by meiotic I non-disjunction, including one case of mosaic trisomy 22 and three cases of mosaic trisomy 16; Four cases arose by meiotic II non-disjunction, including one case each of mosaic trisomy 18 and 22 and two cases of mosaic trisomy 21. Surprisingly, specific recombination hotspots were identified in 16p12.3 and 16q22.1, indicating that these loci are highly prone to cross-exchange.

Six cases with mosaic double trisomies were identified in our study. All cases resulted in intrauterine fetal death during the first trimester, indicating that these mosaic double trisomies are incompatible with embryonic development. Mosaic double trisomies are present in 0.21%~2.8% of early spontaneous abortions, which represent a selective pressure against embryonic development [21, 22]. The lethality of double aneuploidies depends on the specific chromosomes involved, and in certain cases, liveborns have aneuploidies involving chromosomes 8, 13, 18, 21, X, and Y [23]. The mechanism underlying mosaic double trisomies is intricate; the origin of the two chromosomes may be identical or distinct [24, 25]. Here, two cases were from mitotic errors where the mistakes occurred simultaneously with two chromosomes having identical mosaic frequency. Conversely, in the remaining four cases, errors occurred successively with varying mosaic frequencies for different chromosomes. These cases suggest that the assumption of equal mosaic frequency in double trisomies arise from the same mechanisms. On the contrary, if double trisomies arises from different mechanisms, their mosaic frequency are likely to differ.

## Conclusion

We identified 44 cases of mosaicism by the use of SNP array analyses. The utilization of SNP arrays allows for the characterization of mosaicism and provides valuable data for estimating disease mechanisms and recurrence. It is recommended to employ a combination of different technologies for detecting mosaicism.

## Methods

### Study subjects

This study was approved by the institutional research ethics committee of Wenzhou Central Hospital. All parents agreed to participate in the study and provided written informed consent. A total of 4512 pregnant women were referred for genome-wide SNP array testing for prenatal diagnosis at our prenatal diagnosis center in Wenzhou, China, between 2012 and 2018. All pregnant women underwent CVS, amniocentesis, or venipuncture, one of the three options. The indications for prenatal diagnostic testing included: advanced maternal age, high-risk serological screening, abnormal non-invasive prenatal DNA test, abnormal ultrasonographic indications, mother/father carrying chromosomal structural rearrangement, history of intrauterine fetal death or aborted fetuses, and other necessary situations. Peripheral venous blood was collected from the parents if necessary.

### SNP array analysis

DNA was extracted from villi, amniotic fluid, or cord blood. Chromosomal microarray analysis was performed using the Illumina Human CytoSNP-12 array, according to the manufacturer’s instructions. The results were analyzed with Illumina BeadStudio software.

Mosaic changes were detected by assessing for aberrations in probe intensities (log R ratios) along with shifts in genotype frequencies of the SNP probes (B allele frequencies) [26]. Mosaic trisomy is diagnosed when the log R ratio shows an increase in copy number, with between two and three copies; in addition, the B allele frequency must appear to be altered. In the case of mosaic monosomy, the log R ratio indicates a decrease in copy number, between one and two copies.

### Karyotype analysis

Samples of CVS, amniotic fluid, and cord blood were cultured and conventional G-banded karyotyping, according to the standard methods. Karyotypes were analyzed by two physicians, according to the International System for Human Cytogenetic Nomenclature (ISCN) 2020 standard. Additionally, we conducted a comparative analysis of SNP array and conventional karyotype results for each mosaic case.

## Data Availability

All data generated or analyzed during this study are included in the article.

## Abbreviations

CPM: Confined placental mosaicism
TFM: True fetal mosaicism
SNP: Single nucleotide polymorphism
CVS: Chorionic villus sampling
sSMCs: Small supernumerary marker chromosomes
ISCN: International System for Human Cytogenetic Nomenclature
